# Integrating the technology acceptance model for social media-based learning with authentic leadership development: symmetric and asymmetric modeling

**DOI:** 10.3389/fpsyg.2023.1131133

**Published:** 2023-11-13

**Authors:** Muhammad Zaheer Asghar, Samma Faiz Rasool, Pirita Seitamaa-Hakkarainen, Seema Arif, Sumaira Bano

**Affiliations:** ^1^Department of Teacher Education, University of Helsinki, Helsinki, Finland; ^2^Learning and Educational Technology, Faculty of Education and Psychology, Oulu University, Oulu, Finland; ^3^College of Business Administration, Al-Yamamah University, Riyadh, Saudi Arabia; ^4^Department of Education, University of Management and Technology, Lahore, Pakistan

**Keywords:** authentic leadership development, COVID-19, social media-based learning, symmetrical and asymmetrical modeling, technology acceptance model

## Abstract

**Introduction:**

The growing trend of social media use has influenced all segments of society, including education, during the COVID-19 pandemic. At the same time, authentic leadership is an emerging concept in positive psychology for dealing with emergencies in the digital era. The possibility of a relationship between the two was checked in preservice teachers studying in a teacher education department of a university in Pakistan. The conceptual framework of the study was built around teaching acceptance model (TAM) and Authentic leadership theory.

**Methods:**

The survey method suited the aim of the research, and questionnaires aided us in gathering participant self-reporting responses. We conducted two surveys, and in between them, a course was taught online using social media as a teaching and learning platform. The survey results checked preservice teachers’ positive intentions toward social media-based learning, and the post-course survey studied the development of Authentic leadership attributes among the preservice teachers. Technology acceptance was measured across three constructs: ease of use, usefulness, and positive intentions. The results also reported the development of three authentic leadership characteristics: ethics, self-regulation, and self-awareness.

**Results and discussion:**

This study is among the pioneering studies integrating TAM (i.e., the acceptance of social media-based learning) with leadership theory (i.e., authentic leadership). It also adds a methodological contribution by combining symmetrical (i.e., partial least squares structural equation modeling) and asymmetrical (i.e., fuzzy set qualitative comparative technique) for data analysis. The study’s findings are valuable for teacher education institutions, as they help prepare future teachers to become authentic leaders capable of addressing future crises by leveraging education through social media-based teaching and learning platforms.

## 1. Introduction

Higher education has undergone a paradigm shift in learning approaches over the last 2 years. Nearly all educational institutions suspended face-to-face learning processes and transitioned to remote learning through online resources or social media from March 2020 to curb the spread of COVID-19. Traditional universities mostly lacked accessible digital learning resources (Kundu and Bej, 2021). Consequently, social media platforms like WhatsApp, Zoom, YouTube, and Google Meet were extensively employed to facilitate online classes (Asghar et al., 2021). The formal education system remained closed for face-to-face learning during the 2019–2020 academic year across much of the world. However, the formal education system gradually reopened during 2021, with new technologies, such as social media, becoming the new norm. Preservice teachers, being both higher education students and torchbearers of cultural and technological transformation for the next generation, play a vital role. Training preservice teachers for social media-based learning during emergencies has a lasting impact on preparing a generation of teachers equipped with leadership qualities. These teachers are not only capable of handling the current emergencies but also possess the skills to navigate various types of emergencies in the future. This study delves into social media-based learning and its influence on the development of authentic leadership among preservice teachers, particularly in the context of developing countries like Pakistan.

The concept of authentic leadership emerged after the 9/11 incident and with the onset of the dotcom era in the early 2000s (Gardner et al., 2005). The emerging field of positive psychology, along with the concept of authenticity, emphasizes the ownership of personal ideas and experiences (George, 2003). The construct of authentic leadership has been widely discussed in research over the past decade. A systematic literature review conducted by Gardner (2011), ranked as the third most cited paper in The Leadership Quarterly during the last decade, highlighted the significance of the emerging concept of authentic leadership. Researchers have presented the concept of authentic leadership as the development of positive, ethical, and genuine qualities in leaders to effectively navigate complex situations (Iszatt-White et al., 2019). Therefore, it is considered a highly suitable concept for teachers to adopt as authentic leaders, particularly in managing crises such as the COVID-19 pandemic. The digital era has further evolved to encompass the extensive use of social media, especially during the COVID-19 pandemic.

Last year, 2022 saw a marked change in global count of social media users, rising to 4.62 billion. It is a phenomenal change, marking a 10% rise in social media users worldwide (Kemp, 2021a,b). Pakistan has 71.70 million social media users, accounting for 32% of its total population. The Government of Pakistan decided to go online for higher education because of the thick presence of internet networks and a substantial number of social media users. Still, a crisis was in the air; people believed social media to be an entertaining forum, not a teaching and learning platform. The rationale for this research stems from several factors: firstly, while studies exist on the acceptance of online learning (Asghar et al., 2021) and social media-based learning (Dumpit and Fernandez, 2017) in typical scenarios, there is a shortage of research concerning the acceptance of social media as a teaching and learning platform, and a sudden switch to it during the pandemic. Secondly, leadership development research is also embedded predominantly in face-to-face learning environments (Daniëls et al., 2019). Although researchers have examined the amalgamation of technology acceptance theory with leadership theory via literature reviews, empirical substantiation is limited in assessing the impact of social media-based learning on leadership development (Liu and Tao, 2022; Sophea et al., 2022). Thirdly, preceding technology acceptance studies have yielded symmetrical data analysis results lacking comprehensive insights. Fuzzy set comparative qualitative analysis (fsQCA), an asymmetric data analysis technique, has historical roots (Rasoolimanesh et al., 2021) and is gaining traction in management and marketing research. There exists a demand to augment symmetrical data analysis through asymmetrical counterparts in interdisciplinary fields like educational sciences, social media studies, and leadership research.

Fuzzy set qualitative comparative analysis (fsQCA) has emerged as a valuable instrument for analysis in social sciences research (Asghar et al., 2023). Rooted in set theory and Boolean algebra, fsQCA facilitates the exploration of multifaceted causal configurations when numerous conditions interact to yield outcomes (Pappas and Papatheodorou, 2017). This study endeavors to harness the efficacy of fsQCA in scrutinizing the interplay between authentic leadership and the acceptance of technology for social media-based learning, particularly among preservice teachers grappling with the exigencies of the COVID-19 pandemic. Such a configurational analysis promises insights into the diverse pathways (Ragin, 2008) through which social media-based learning impacts the development of leadership attributes among preservice teachers, equipping them to effectively address educational crises. Additionally, fsQCA’s capacity for contextual exploration, combination analysis, configurational assessment, and theory advancement augments comprehension (Rasoolimanesh et al., 2021) of the interconnected relationship between technology acceptance theory and leadership development in times of crisis. It offers a distinct methodological contribution to knowledge enrichment within the interdisciplinary domain of information sciences, leadership development, educational sciences, and crisis management.

The aim of this study is to investigate the impact of social media-based learning on the development of authentic leadership among preservice teachers in the context of developing countries, such as Pakistan. This study is the first of its kind to concurrently examine social media-based learning, the technology acceptance model for social media-based learning, and authentic leadership development theory in the context of developing countries. This study contributes to knowledge in several ways. Firstly, it explores the direct relationship between perceived use, perceived ease of use, and the use of social media for fostering authentic leadership development. Secondly, it delves into the direct connection between perceived use, perceived ease of use, and intentions toward social media-based learning. Thirdly, it investigates the direct correlation between intentions toward social media-based learning and its application in cultivating authentic leadership development. Fourthly, it tests the mediating role of intentions toward social media-based learning in the relationship between perceived use, perceived ease of use, and social media utilization for authentic leadership development. Additionally, this study introduces a novel approach by employing both symmetrical and asymmetrical methodologies for rigorous statistical analysis–partial least squares structural equation modeling and qualitative comparative analysis (i.e., fuzzy sets qualitative comparative analysis).

This study holds significance for educational institutions, higher education establishments, and government-level educational policymakers as it offers insights for designing and implementing social media-based instructional strategies aimed at nurturing teachers’ development as authentic leaders. Drawing from the existing literature and the discussions presented in this study, two research questions (RQs) are proposed:

RQ1: Does social media-based learning influence the development of authentic leadership among preservice teachers?

RQ2: Does the technology acceptance model for social media-based learning align with authentic leadership development theory?

The structure of this paper is organized as follows: The first part introduces the research problem, the second section elucidates the theoretical and conceptual framework; similarly, the third and fourth sections detail the research methods. In the third section, we delve into the findings and analysis derived from this study. Finally, the concluding section encompasses theoretical and practical implications, limitations, and potential directions for future research.

## 2. Theoretical framework

This study is founded upon the theoretical framework of the technology acceptance model (TAM) (Davis, 1989) and authentic leadership theory (George, 2003). The research encompasses four distinct stages that outline the hypothetical correlations among variables. Firstly, the technology acceptance model (TAM) is employed. The exogenous variables of TAM, namely, perceived use (PU) and perceived ease of use (PEU), are postulated to have hypothetical correlations with social media usage for fostering authentic leadership development. Secondly, both PU and PEU are hypothesized to correlate with intentions toward engaging in social media-based learning. Thirdly, there exists a hypothetical correlation between intentions for social media-based learning and its application in authentic leadership development. Finally, it is proposed that intentions toward social media-based learning serve as a mediating factor between PU, PEU, and the utilization of social media for authentic leadership development.

### 2.1. Technology acceptance model

The technology acceptance model (TAM) is a theoretical framework developed by Fred Davis in the late 1980s (Davis, 1989) that explains why people accept or reject a new technology (Na et al., 2022). The TAM’s framework profoundly influences researchers and practitioners to understand user behavior for technology acceptance (Surendran, 2012). The TAM has become more comprehensive after including external factors and social-psychological influences in different contexts (Vuković et al., 2019). Focused on user-centric technology design, TAM has gained popularity with its diverse applications in software development, online learning platforms, and other virtual environments (Zaineldeen et al., 2020). It enhances experiences and promotes successful adoption of new technologies. This model describes the cognitive processes forming our technological choices, enabling a pathway to boost wider acceptance of new innovations. The technology acceptance model (TAM) (Davis, 1989) consists of three dimensions, i.e., (i) perceived ease of use, (ii) perceived usefulness, and (iii) intentions to use.

#### 2.1.1. Perceived ease of use (PEU)

PEU indicates the level of comfort associated with utilizing social network platforms (Magsamen-Conrad et al., 2020). The impact of information technology on human life is immense, and its significance in education remains indisputable (Surendran, 2012; Vuković et al., 2019). Particularly in the context of the COVID-19 pandemic, information technology’s influence has surged due to the closure of educational institutions, posing challenges for students. Particularly, easy access to study resources has emerged as a prominent factor driving students’ inclination toward social media-based learning (Wijesundara and Xixiang, 2018; Alsaleh et al., 2019; Alshurideh et al., 2019). This trend underscores the role of social media tools in facilitating convenient information access, thereby fostering positive intentions among students. Moreover, it profoundly influences learners’ behavior in adopting social media-based learning for the development of authentic leadership skills.

#### 2.1.2. Perceived usefulness (PU)

Perceived usefulness defines the level at which scholars believe using social networks will help maintain their learning activities (Jacob and Pattusamy, 2020). In the last few years, social media has spread across the globe. Being a substantial and integral part of today’s everyday life, many people, especially on online social networks, have fundamentally changed their communication behavior. Unfortunately, not everybody is integrated into this “new everyday life” yet. Social media usage in education is on the rise as an emerging economy. Previous studies have found a positive influence of perceived usefulness (PU) on intentions and actual usage of social media for learning (Doleck et al., 2017; Ifinedo, 2018; Alsaleh et al., 2019). The virtual environmental characteristics, such as collaboration, communication, and resource sharing, facilitated by social media adoption within the academic community during COVID-19, have also increased due to the efficient way social media connects with other people (Popescu and Badea, 2020; Liu et al., 2022). This is the reason that learners’ perceived usefulness of social media influences their intentions toward social media-based learning. It also affects learners’ actual behavior in using social media-based learning for authentic leadership development.

#### 2.1.3. Intentions to use (INT)

Intentions involve predicting certain behaviors, such as using social media for learning (Asghar et al., 2021). The application and usefulness of social media extend to resource sharing and interaction with academics in educational institutions beyond physical boundaries (Asghar et al., 2022). Furthermore, engaging with educators, learners, and engaging in online information-sharing behavior significantly impacts students’ engagement and academic performance (Asghar et al., 2021). Social media, when used for collaborative learning, empowers students to be more creative, dynamic, and research-oriented (Asghar et al., 2022). This is the reason why previous studies have demonstrated a positive effect on learners’ intentions to use social media for learning (Acarli and Sağlam, 2015; Behringer and Sassenberg, 2015; Ifinedo, 2018). We examined the influence of preservice teachers’ intentions toward social media-based learning on their utilization of social media for authentic leadership development.

### 2.2. Authentic leadership development (ALD)

Authentic leadership is a concept with its origins in Ancient Greek history. Various researchers have presented different facets of authentic leadership from the perspective of positive psychology (Voshel and Wesala, 2015; Nair et al., 2021; Steffens et al., 2021; Khan and Ghayas, 2022). According to this concept, a genuine leader needs to possess four essential characteristics (Hendriks et al., 2020). The first is prudence; the leader must consider all matters impartially. The second is temperance, requiring the leader to maintain emotional balance and control. The third is justice, which entails handling all matters fairly. The fourth is fortitude, signifying a leader’s courage to fearlessly do what is right. These characteristics aid a leader in enhancing their inner self and relationships with others.

Authentic leadership emerged as a topic of theoretical discourse in the late 1960s, primarily driven by the rise of certain detrimental elements. George (2003) authored a book on “Authentic Leadership” in 2003, in which he depicted authentic leaders as individuals committed to organizational development, possessing a profound sense of purpose, and remaining true to their core values. George (2003) argues that leadership is not an innate trait but a product of time and effort. He also outlines three phases in the journey of becoming a leader. The first phase involves mental preparation for the journey, as becoming an authentic leader is a lengthy process. The second phase necessitates embracing new challenges and offering solutions to difficulties, ultimately reaching the pinnacle of leadership. This phase entails sharing knowledge and wisdom to address societal issues and contributing to the community. The learning process continues. Leadership is not a self-proclaimed status; it is about demonstrating authenticity and wisdom through one’s actions.

#### 2.2.1. Social media and the pillars of authentic leadership

Authentic leadership comprises three fundamental pillars: awareness, self-regulation, and internalized moral perspective (Avolio and Gardner, 2005; Beddoes-Jones and Swailes, 2015). We have operationalized these three pillars of authentic leadership concerning the utilization of social media-based learning for authentic leadership development.

##### 2.2.1.1. Self-regulation

Gardner et al. (2005) defined self-regulation as an individual’s ability to uphold moral values independently, unaffected by group, organizational, or societal influences. Self-regulation is another crucial facet of authentic leadership, exemplified by relational transparency, which involves self-discourse, openness, and trust in close relationships. Prior research supports a connection between social media-based learning and students’ self-regulation (Wolf et al., 2017; Alaimo and Kallinikos, 2019). According to Zimmerman (1990), self-regulation is a learning outcome that guides students to engage in various educational activities, set goals, identify sources to achieve these goals, and assess the resulting learning outcomes. The use of social media enhances self-regulation among learners through collaborative learning experiences (McLoughlin and Lee, 2007), and teachers’ integration of social media into classroom instruction complements self-regulation. Additionally, the assumption is that social media-based learning can enhance educational outcomes by facilitating learners’ self-regulation (McLoughlin and Lee, 2010). Thus, we apply the concept of social media-based learning and self-regulation within the context of authentic leadership, with implications for fostering students’ self-regulation in virtual environments, maintaining trust, relational transparency, openness, and disclosure in relationships with followers.

##### 2.2.1.2. Ethical aspects

Assiri (2018) demonstrates this across personal and professional relationships and decision-making. Furthermore, the moral perspective is not externally dictated; rather, it should emanate from an authentic leader’s internalized moral values. Ethical leaders exhibit unwavering consistency in actions and interactions (Stoller, 2013), and leaders in social media environments must consider ethical considerations for effective outcomes. According to Demers and Sullivan (2016), dignity, confidentiality, and respect are integral components of ethical behavior in social media environments, and leaders must adeptly lead in virtual settings. The intricacies of social media, encompassing both immediate and enduring impacts of activities, necessitate ethical mindfulness (Voshel and Wesala, 2015). This is reflected in the ethical standards upheld by platforms like Facebook, YouTube, Twitter, and WhatsApp (Warnick et al., 2016). As students engage with these platforms, they learn from interactions and mistakes (White and Boatwright, 2020). The ethical dimension of leadership is uncompromising, devoid of situational or external influences (Johnson, 2015). Ethical standards apply to ordinary social media users, making it imperative for leaders to grasp ethical practices in virtual realms. This fosters the development of a reputation that mirrors behavioral consistency. Researchers (Kietzmann et al., 2012) emphasize that leaders’ ethical traits play a pivotal role in reputation development based on past interactions with their followers (Duh and Dabula, 2021).

##### 2.2.1.3. Self-awareness

Authentic leadership embodies self-awareness of actions and reactions (Corriveau, 2020) and acknowledging limits. Self-awareness entails comprehending personal strengths, weaknesses, and one’s nature in relation to the world (Khan and Ghayas, 2022). It also encompasses understanding one’s emotions, emotional stability, and motivation, providing insights into the universe and its surroundings. Such decisions result in mutual benefit and create win-win situations (Bryan and Vitello-Cicciu, 2022). Self-awareness within authentic leadership can manifest in various ways (Balogun et al., 2020), including recognizing strengths and weaknesses, appreciating the multi-layered nature of the self, learning about one’s impact on others and vice versa, and fostering ongoing self-exploration and development (Nair et al., 2021; Steffens et al., 2021). According to Tuten et al. (2015), communication strategies aid in exchanging ideas and delivering value to learners, elevating awareness of learners’ positions within an educational organization. In this vein, social media-based learning offers students a platform for self-awareness and self-realization. Such sources of social media-based learning may encompass Facebook, Twitter, YouTube, and Instagram (Tuten and Mintu-Wimsatt, 2018).

### 2.3. Conceptual framework

**Figure 1:**
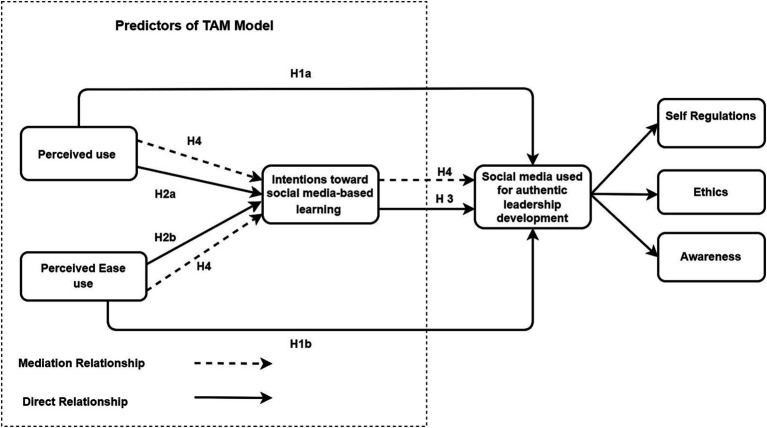
Conceptual framework.

The above-discussed conversation led us to propose PEU (Perceived ease of use) and PU(perceived usefulness) as exogenous constructs for INT (intentions). Meanwhile, INT is suggested to be an exogenous construct for SMU_INT (Social media use and intention formation), as illustrated in Figure 1. This study encompasses four hypotheses. Firstly, it investigates the positive and significant direct correlation between perceived use (H1a) and perceived ease of use (H1b) with social media’s utilization for authentic leadership development. Secondly, we test the positive and significant direct relationship between PU (H2a) and PEU (H2b) with intentions toward social media-based learning. Thirdly, this study explores the direct connection between intentions toward social media-based teaching and learning-based platforms and social media’s application for authentic leadership development (H3). Fourthly, we examine the mediating effect of intentions toward social media-based learning between perceived use (H4), perceived ease of use, and the utilization of social media for authentic leadership development. The solid lines within the framework represent direct relationships, while the dotted lines signify mediating relationships.

## 3. Method

This study has employed survey method for two reasons. Firstly, investigating students’ intentions to use social media for learning and its impact on their usage behavior necessitates a self-reported survey methodology. Secondly, utilizing a survey enables researchers to gain insights from a diverse audience, contributing to the generalizability of results across a wide population of preservice teachers. In this way, survey method suited the objectives of the study.

### 3.1. Questionnaire development

The questionnaire is divided into four parts. The first part introduces the study’s purpose. The second part includes demographic information. The third part encompasses constructs based on the technology acceptance model (TAM), consisting of four main constructs: “perceived ease,” “perceived ease of use,” “attitude,” and “behavioral intentions.” These constructs’ items were adopted from existing questionnaires (Venkatesh et al., 2003; El-Gayar and Moran, 2006; Yurdakul et al., 2014) and rated on a Likert-type scale ranging from “1 = strongly disagree” to “5 = strongly agree.”

The second questionnaire also contained similar three parts as the questionnaire A. The fourth part comprises constructs related to “authentic leadership development,” encompassing three main constructs: “self-awareness,” “ethics,” and “self-regulation.” Items for these constructs were adopted from previous research (Avolio and Gardner, 2005; Bertoncini and Schmalz, 2013) and were also rated on a Likert-type scale ranging from “1 = strongly disagree” to “5 = strongly agree.”

A pilot test was conducted initially, involving 20 PhD students and 10 professors who completed the surveys. The results of the pilot test demonstrated the questionnaire’s reliability and validity.

#### 3.1.1. Construct measurement

The construct “perceived use” comprises three items selected from prior studies (Venkatesh et al., 2003; El-Gayar and Moran, 2006; Yurdakul et al., 2014). These items are as follows: “I find social media for learning useful,” “Using social media enables me to complete academic tasks more efficiently,” and “Using social media enhances the effective utilization of my time in managing academic tasks.” A Cronbach’s alpha value exceeding the threshold of 0.7 indicates satisfactory construct reliability (Hair et al., 2013). The pilot survey demonstrated satisfactory construct reliability with *α* = 0.717.

The construct “perceived ease of use” consists of three items drawn from previous research (Venkatesh et al., 2003; El-Gayar and Moran, 2006; Yurdakul et al., 2014). These items include: “My interaction with social media for learning is clear and comprehensible,” “I am adept at using social media for learning,” and “Learning to use social media for learning is straightforward for me.” A Cronbach’s alpha value surpassing the threshold of 0.7 reflects satisfactory construct reliability (Hair et al., 2013). The pilot survey showed satisfactory construct reliability with *α* = 0.852.

The construct “intentions toward social media-based learning” encompasses seven items extracted from prior studies (Venkatesh et al., 2003; El-Gayar and Moran, 2006; Yurdakul et al., 2014). Sample items include: “I intend to utilize social media for educational purposes,” “I plan to use social media tools (such as Zoom, Monkey Survey, and WhatsApp) for learning,” and “I aim to employ social media to connect with my teachers, mentors, and classmates.” A Cronbach’s alpha value exceeding the threshold of 0.7 indicates satisfactory construct reliability (Hair et al., 2013). The pilot survey demonstrated satisfactory construct reliability with *α* = 0.872.

The primary construct of using social media for authentic leadership development comprises three sub-factors: self-awareness, ethics, and self-regulation.

For the construct “self-awareness,” an item was selected from the work of Beddoes-Jones and Swailes (2015): “The use of social media for learning has prepared me as a leader, I [...]: consistently put myself in others’ shoes and perceive situations from their perspective, remain conscious of my emotions, beliefs, and motives, leverage my experiences as opportunities for self-discovery, remain attuned to my emotions to understand their influence on me, and recognize how my moods and actions impact others.” A Cronbach’s alpha value exceeding the threshold of 0.7 indicates satisfactory construct reliability (Hair et al., 2013). The pilot survey demonstrated satisfactory construct reliability with *α* = 0.832.

For the construct “self-regulation,” items were selected from the work of Beddoes-Jones and Swailes (2015): “The use of social media for learning has prepared me as a leader, I [...]: maintain control over my ego, avoid fluctuations in mood, sustain approachability even in the face of significant challenges, and consistently exemplify behavior for others to follow.” A Cronbach’s alpha value exceeding the threshold of 0.7 indicates satisfactory construct reliability (Hair et al., 2013). The pilot survey showed satisfactory construct reliability with *α* = 0.726.

For the construct “ethics,” an item was selected from the work of Beddoes-Jones and Swailes (2015): “The use of social media for learning has prepared me as a leader, I [...]: engage in discussions about challenging ethical matters with others, perceive ethics as an active choice rather than a compromise, remain resolute in my ethical stance despite dissent from others, and maintain clarity regarding my core values.” A Cronbach’s alpha value exceeding the threshold of 0.7 indicates satisfactory construct reliability (Hair et al., 2013). The pilot survey demonstrated satisfactory construct reliability with *α* = 0.746.

### 3.2. Data collection strategy

We collected data from students in two separate time slots. Initially, 405 participants completed an online survey in June 2020. The first survey gauged students’ intentions regarding using social media as a teaching and learning platform.

Subsequently, the second survey was conducted 4 months after the implementation of social media-based learning strategies. This follow-up survey assessed the influence of social media-based learning on students’ utilization of social media for fostering awareness, ethics, and self-regulation as authentic leaders. In this phase, 396 students participated in the survey. Overall, we included 97% of the respondents from the first survey to gather opinions in the second.

The average age of the research participants was 21.5 years. Among them, 69% identified themselves as female, 30% as male, and 1% preferred to remain unidentified. Furthermore, 98% of the respondents were enrolled in graduate programs, and the remaining 2% were in postgraduate programs. The participants were proportionally distributed across various fields of specialization, including special needs education (19.2%), educational leadership and management (19.5%), elementary education (21.9%), and educational sciences (39.5%).

## 4. Data analysis

We used two parallel techniques, symmetrical (SmartPLS) and asymmetrical (fsQCA), for data analysis. Rasoolimanesh et al. (2021) recommend integrating the analytical power of SmartPLS and fsQCA to facilitate an in-depth exploration of the interplay of research variables. SmartPLS is a robust tool that enables us to explore the association within the study model comprehensively (Hussain et al., 2023). A structural equation model (SEM) was obtained using this tool, providing insight into the complex relationships between variables. Thus, we could examine the direct and indirect relationships among constructs in the model. fsQCA, a method rooted in the principles of qualitative comparative analysis, was employed in addition to SmartPLS. It has already been successfully applied in a similar study by Asghar et al. (2023). The successive use of the tools helped us dig deeper into the intricate patterns and configurations in the variables (Ragin, 2008). Results thus obtained have identified critical relationships among constructs enriching the outcomes and future research possibilities in this avenue.

### 4.1. Measurement model

It was imperative to assess the outer model’s reliability, consistency, and validity to validate the factors. Accepting item loadings above the threshold of 0.4 (Hair et al., 2013) maintained the desired single-item consistency with their respective constructs. All individual items demonstrated a satisfactory level of loading, ranging from 0.733 to 0.904. Studies (Hair et al., 2011; Rasool et al., 2020) have recommended α, rho-alpha, and composite reliability (CR) values exceeding the threshold of 0.7 to establish reliable constructs. Cronbach’s alpha values, ranging from 0.717 (perceived use) to 0.872 (intentions), showcased satisfactory consistency and reliability of the constructs, as shown in Table 1. The average variance explained (AVE) signified the divergent validity of the constructs. An AVE value surpassing the threshold of 0.5 indicates a satisfactory level of divergent validity (Zhou et al., 2021). The AVE of the constructs in this study ranged from 0.565 (intentions) to 0.696 (perceived ease of use), indicating a satisfactory level of divergent validity (see Table 2). The Cronbach’s alpha, rho-alpha, and CR values for the second-order Authentic Leadership (ALD) factor also indicated satisfactory consistency and reliability. Additionally, the AVE value for ADL exceeded the threshold of 0.5, as indicated in Table 1.

**Table 1:** Reliability and construct validity.

Factors	Items	Loading	α	rho_A	CR	(AVE)
Intentions	BI1	0.733	0.872	0.872	0.901	0.565
	BI2	0.736				
	BI3	0.772				
	BI4	0.795				
	BI5	0.743				
	BI6	0.738				
	BI7	0.744				
Perceived ease of use	PEU1	0.853	0.852	0.854	0.901	0.696
	PEU2	0.879				
	PEU3	0.729				
	PEU4	0.868				
Usefulness	PU1	0.843	0.717	0.717	0.819	0.605
	PU2	0.650				
	PU3	0.825				
Self-awareness	AS1	0.793	0.832	0.837	0.882	0.599
	AS2	0.800				
	AS3	0.816				
	AS4	0.772				
	AS5	0.683				
Ethics	ET1	0.733	0.746	0.746	0.84	0.567
	ET2	0.78				
	ET3	0.761				
	ET4	0.738				
Self-regulation	SR1	0.801	0.726	0.726	0.845	0.646
	SR2	0.789				
	SR3	0.821				
Second-order factor						
Intentions to use social media	Ethics	0.904	0.872	0.876	0.921	0.796
	Awareness	0.893				
	Self-regulation	0.880				

**Table 2:** HTMT for first-order factors.

Constructs	Awareness	Ethics	Intentions	Perceived ease of use	Perceived use	Self-regulation
Awareness						
Ethics	0.839					
Intentions	0.809	0.747				
Perceived ease of use	0.677	0.613	0.680			
Perceived use	0.742	0.618	0.818	0.646		
Self-regulation	0.806	0.898	0.708	0.546	0.637	

#### 4.1.1. Convergent validity

Henseler (2012) introduced a distinct measure known as the heterotrait–monotrait ratio of correlations (HTMT) to evaluate the discriminant validity of constructs. An HTMT ratio below the threshold of 0.9 indicates the absence of multicollinearity issues (Sarstedt et al., 2014). All constructs under scrutiny exhibited HTMT ratios below the specified threshold of 0.9, as depicted in Table 2.

The first-order factors, namely, self-awareness, ethics, and awareness, constitute the second-order factors of ALD. The HTMT ratios for the second-order ALD factor similarly indicate the absence of multicollinearity concerns for all constructs, as evidenced in Table 3.

**Table 3:** HTMT ratios for second-order factors.

Constructs	Intentions	Perceived ease of use	Perceived use	Use behavior
Intentions				
Perceived ease of use	0.68			
Perceived use	0.818	0.646		
Authentic leadership development	0.803	0.665	0.697	

### 4.2. Structural equation model evaluation

Following the evaluation of construct reliability and validity, a four-step procedure was employed to assess the structural equation modeling. These steps encompassed measurements of multicollinearity, model fit indices, goodness of fit for the model, determination of the coefficient of determination (*R*^2^), effect size analysis, redundancy analysis, and assessment of direct and specific indirect paths.

#### 4.2.1. VIF stats

The variance inflation factor (VIF) indicates the degree of multicollinearity among constructs. Researchers have recommended that VIF values remain below the threshold of 0.5 (Hair et al., 2011; Rasool et al., 2021). The VIF values in this study were consistently below the suggested threshold (<0.5), indicating the absence of collinearity issues, as depicted in Table 4.

**Table 4:** VIF statistics.

Constructs	Intentions	Perceived use	ALD	Model fit
Intentions			1	SRMR = 0.063 NFI = 0.81
Perceived ease of use		1		
Perceived usefulness	1.313			

The standardized root mean squared residual (SRMSR) and the normed fit index (NFI), also known as the Bentler and Bonett Index, are key indicators of model fitness in PLS-SEM (Chen, 2007; Samma et al., 2020). The standardized root mean residual (SRMR) is a standardized residual reflecting the ratio between covariance matrices of hypotheses and observed covariance matrices. The NFI assesses the discrepancy between the chi-square of the target structural equation model (SEM) and the null model, with the result divided by the chi-square of the null model. As per Hu and Bentler (1998), an SEM with an SRMR below the threshold of 0.08 and an NFI value above 0.8 is deemed to exhibit good fit. In this study, the values of SRMR (0.063) and NFI (0.81) meet the established threshold criteria, as presented in Table 4.

#### 4.2.2. Structural model path coefficients

Mean values in PLS-SEM resemble the β coefficients used in regression analysis (Grimsey and Lewis, 2002). The β coefficient signifies the change in dependent variables resulting from a unit change in the independent variable. The significance level gauges the validity of hypothesis testing, which can also be measured using the t-test. In this study, mean values were evaluated as β coefficients, *t*-test statistics, and significance values for verification through 5,000 bootstrapped sub-samples.

Perceived ease of use exhibited a positive and significant impact on social media use for authentic leadership development (*M* = 0.228, *t* = 5.385, *p* < 0.001), thereby confirming hypothesis 1a. Additionally, a positive and significant relationship between perceived ease of use and perceived usefulness of social media plateform for authentic leadership development was observed (*M* = 0.141, *t* = 2.811, *p* < 0.001), leading to the acceptance of hypothesis 1b. Data analysis revealed a positive and significant correlation between perceived ease of use and intentions (*M* = 0.342, *t* = 8.172, *p* < 0.001), solidly supporting hypothesis 2a. Furthermore, a significantly positive relationship between perceived usefulness and intentions was observed (*M* = 0.488, *t* = 10.444, *p* < 0.001), resulting in the acceptance of hypothesis 2b.

Intentions demonstrated a positive and significant influence on social media use for authentic leadership development (*M* = 0.474, *t* = 9.827, *p* < 0.001), thus supporting hypothesis 3. This study demonstrated a robustly positive and significant mediation effect of intentions between perceived ease of use and social media use for authentic leadership development (*M* = 0.162, *t* = 6.169, *p* < 0.001), confirming hypothesis 4a. Similarly, intentions also exhibited a positive and significant mediation effect between perceived use and social media use for authentic leadership development (*M* = 0.227, *t* = 7.757, *p* < 0.001), leading to the acceptance of hypothesis 4b, as presented in Table 5.

**Table 5:** Direct effects.

Hypothesis	Original sample (O)	Sample mean (M)	Standard deviation (STDEV)	T statistics (|O/STDEV|)	*p* values
Perceived ease of use → Intentions	0.354	0.342	0.043	8.172	0.000
Perceived usefulness → Intentions	0.471	0.488	0.045	10.444	0.000
Intentions → SMU_ALD (Social media use_Authentic Leadership)	0.482	0.474	0.049	9.827	0.000
Perceived ease of use → SMU_ALD	0.229	0.228	0.043	5.385	0.000
Perceived use → SMU_ALD	0.131	0.143	0.047	2.811	0.005
Specific in-direct paths					
Perceived ease of use → Intentions →SMU_ALD	0.171	0.162	0.028	6.169	0.000
perceived usefulness → Intentions → SMU_ALD	0.227	0.231	0.029	7.757	0.000

*R*^2^ value Coefficient of determination.

The coefficient of determination, R^2^, illustrates the extent to which variations in the dependent construct can be predicted by changes in the independent construct. Researchers have recommended that the R-square value should exceed the threshold of 0.1 (Hair et al., 2016). Notably, all R-square values for the constructs, including intentions (51%) and social media use behavior for authentic leadership development (54%), surpassed the 10% benchmark, as depicted in Table 6.

**Table 6:** R-square values of study construct.

	R square	R square adjusted
Intentions	0.514	0.512
SMU_ALD	0.545	0.542

Effect size.

As per Cohen (1988), an effect size below 0.02 is considered weak, below 0.15 is moderate, and above 0.35 is substantial. The F-square values in our study indicate that all exogenous constructs exerted a moderate effect on the endogenous constructs, as presented in Table 7.

**Table 7:** F-square values of study construct.

Constructs	Intentions	SMU_ALD
Intentions		0.248
Perceived ease of use	0.193	0.072
Perceived use	0.342	0.021

**Figure 2:**
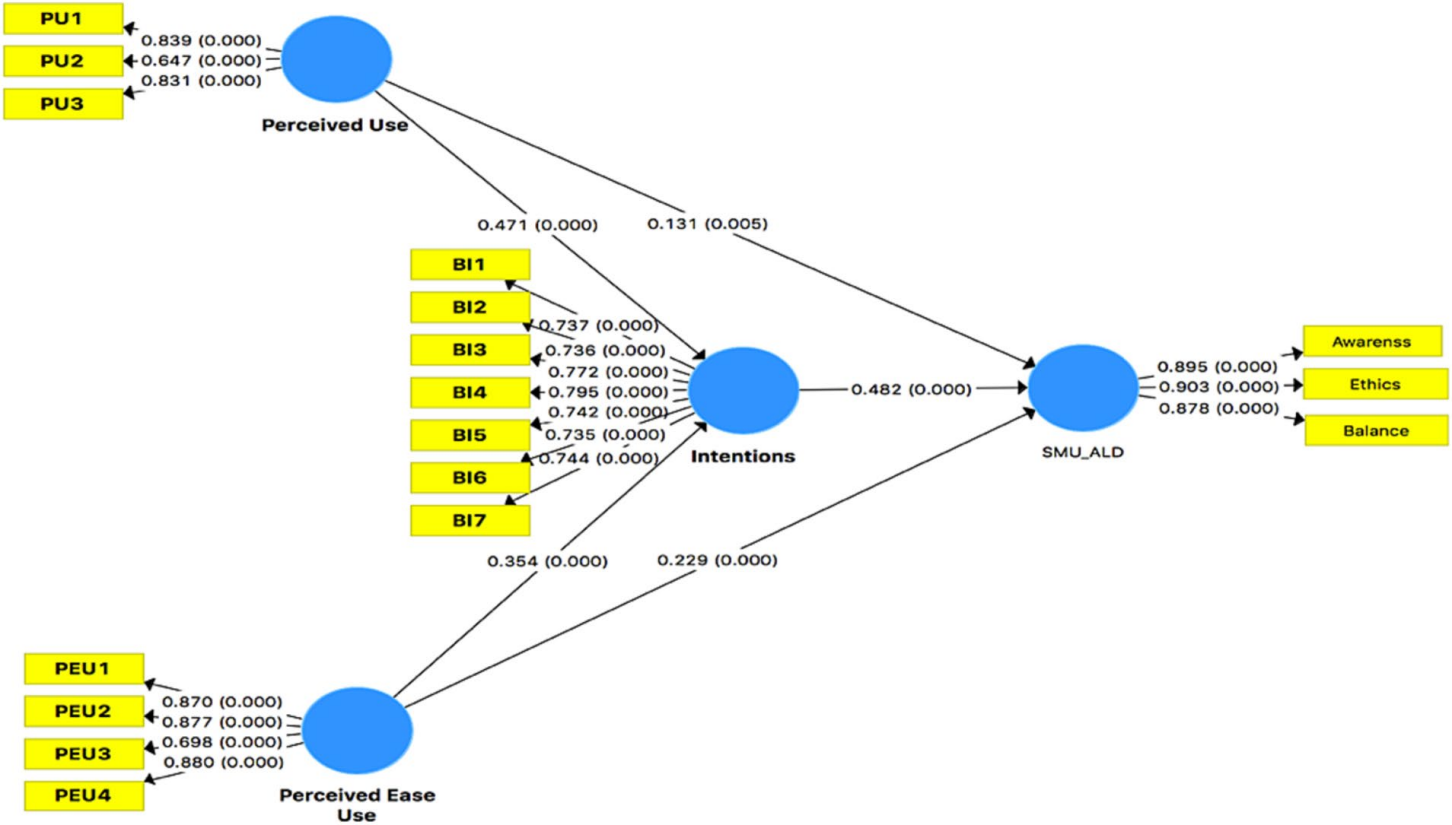
Unified theory of technology acceptance and authentic leadership.

The overall relations and values of the predicted models are shown in Figure 2.

### 4.3. Fuzzy-set qualitative comparative analysis

We retrieved the CSV output file of the PLS-SEM results from SmartPLS, which served as the input for fsQCA. This CSV file contains standardized scores ranging from −3 to 3, which were then calibrated into distinct ranges: −3 to 0, 0 to 0.5, and 3 to 1, as outlined by previous research (Rasoolimanesh et al., 2021).

In accordance with Rubinson (2019), we proceeded by constructing a truth table, a vital step to comprehend the potential combinations of conditions necessary for the study’s desired outcomes. Considering a larger sample size (Fiss, 2011), advised setting the consistency threshold to 3, leading to the removal of rows with fewer than two cases. Following the recommendation by Rasoolimanesh et al. (2021), we identified combinations with a coverage exceeding 0.2 and a consistency level surpassing 0.8.

The fsQCA analysis yielded four types of outcomes: parsimonious, intermediate, complex, and simple solutions. As per Pappas and Woodside (2021), a solution pertains to a combination of configurations supported by a substantial number of cases, where a core combination yields consistent outcomes. The literature advocates selecting intermediate solutions (Olya et al., 2020; Rasoolimanesh et al., 2021), a guidance we adhered to in this study.

The fsQCA results, detailed in Table 8 under the outcome of “social media use for authentic leadership development,” showcased heterogeneous combinations of PU, PEU, and INT dimensions resulting in a high-level SMU_ALD. The outcomes also revealed sufficient configurations generating SMU_ALD. The table presents two configurations: configuration 1 (PU*PEU*) highlights the significance of PU and PEU in driving SMU_ALD, while configuration 2 (~PUINT* ~ PEU) underscores the importance of INT in generating SMU_ALD even when PU and PEU levels are low.

**Table 8:** Configurations for SM_ALD.

Configurations	Raw coverage	Unique coverage	Consistency
Model: Social media use for authentic leadership development = f (PU, INT, PEU)			
PU*PEU	0.967784	0.397499	0.978657
~PU*INT* ~ PEU	0.577492	0.00720733	0.846585
solution coverage: 0.974992			
solution consistency: 0.891104			

Table 9 displays the adequate configurations of PU*PEU that result in the generation of INT.

**Table 9:** Configurations for INT.

Configurations	Raw coverage	Unique coverage	Consistency
Model: Intentions to use social media for learning = f (PU, INT, PEU)			
PU*PEU	0.834329	0.834329	0.849313
solution coverage: 0.834329			
solution consistency: 0.849313			

The descriptive statistics for the constructs are as follows: PU (*M* = 0.51, SD = 0.20), INT (*M* = 0.56, SD = 0.20), PEU (*M* = 0.52, SD = 0.21), and SMU_ALD (*M* = 0.57, SD = 0.211).

**Figure 3:**
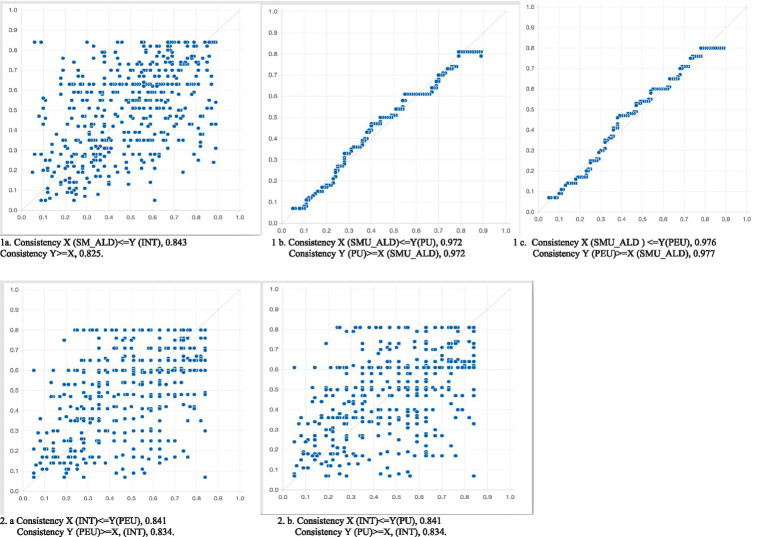
Linear relationships between exogenous and endogenous constructs.

The fsQCA Graphs 1a, 1b, and 1c reinforce the findings of both the PLS-SEM hypotheses and fsQCA configurations. Graph 1a depicts a linear and positive relationship between INT and SMU_ALD. Graph 1b illustrates a linear and positive relationship between PEU and SMU_ALD. Meanwhile, Graph 1c demonstrates a linear and positive relationship between PU and SMU_ALD. In Graph 2a, a positive and linear relationship between PEU and INT is presented, while Graph 2b portrays a linear and positive relationship between PU and INT. These graphical representations are collectively presented in Figure 3.

## 5. Discussion and conclusions

New technological advancement is transforming the learning horizons and resolve most of the current educational issues such as access to education and interactive pedagogy. According to Deffenbaugh (2010), states that Generation Z and millennial raised in a digital era are entering higher education, and their learning needs differ. Technological advancement has affected their learning styles from visual-verbal to virtual spaces (Proserpio and Gioia, 2007). Therefore, it is evident that teachers need to transform their pedagogies to stay authentic (Wankel, 2009). Leadership begets leadership. Mastery of self-awareness, ethics, and self-management is necessary for higher education faculty before commanding it in their students. This reciprocity shall make the whole leadership process authentic, generating authentic educational leadership. We may consider COVID-19 a blessing in disguise as it has initiated this process in students and teachers of Pakistan. The outcomes of the study have numerous theoretical and practical implications.

### 5.1. Theoretical implications

This study contributes theoretically in three significant ways. Firstly, the findings validate the technology acceptance model (TAM) in the context of social media-based learning. Concurrently, prior research (Mohammadi, 2015) has already emphasized TAM’s relevance in e-learning, blended learning, and hybrid learning, signifying its continued applicability in investigating transformative new learning technologies. Our study underscores the pivotal role of perceived ease of use and perceived usefulness as fundamental precursors in shaping learners’ intentions toward social media-based learning.

Secondly, this study bridges two distinct theories: the technology acceptance model and authentic leadership theory. It extends our understanding by revealing that the utilization of social media to enhance learners’ awareness, foster a balanced personality, and cultivate ethical acumen contributes to the development of authentic leaders. Despite higher education institutions’ efforts to impart ethics and foster balanced personalities through traditional means, our research indicates that these teachings often prove inadequate when students confront real-life situations. As highlighted by researchers (Wang and Calvano, 2015), students who have undergone ethics education exhibit similar levels of moral responsibility as those who have not. This discrepancy between leadership theories and their real-world application, as pointed out by researchers (Venkat Raman et al., 2019; Sholihin et al., 2020), underscores the gap in nurturing well-rounded, morally conscious, and self-regulated authentic leaders. This could be attributed to challenges in implementing certain learning activities due to high costs, limited resources, and associated risks (Salas et al., 2009; Loon et al., 2015). In this context, emerging technologies like mobile phones and social media platforms offer an alternative avenue for real-life experiences in the virtual realm, an aspect that is challenging to replicate through traditional pedagogical methods such as books, lectures, and paper-based assessments. These new technologies facilitate enhanced social learning experiences for students, enabling more direct interaction with the real world within the virtual environment.

Thirdly, this research serves as a pioneering effort in the interdisciplinary realm of social media, leadership, and educational sciences by presenting symmetrical and asymmetrical modeling. Previous studies have endorsed the combination of these two approaches in different disciplines (Pappas and Papatheodorou, 2017; Pappas and Woodside, 2021; Rasoolimanesh et al., 2021; Asghar et al., 2023). This methodological innovation underscores the capability of fsQCA to reinforce the outcomes derived from PLS-SEM analysis.

### 5.2. Practical implications

Why are social media tools gaining popularity among young people? As children grow, they tend to explore their self-identity. Identities are shaped by social interaction. Social media tools offer broader opportunities for young individuals to discover themselves and establish boundaries with others. They learn through social media by presenting themselves, forming relationships, engaging with individuals and groups, understanding different perspectives, and managing their privacy and intimacy. “Because identities are constructed within discourse, rather than outside of it, we need to understand them as products of specific historical and institutional contexts within distinct discursive formations and practices, shaped by particular enunciative strategies, and influenced by specific modalities of power” (Hall and Jing, 1996).

Due to the dual nature of self-development, social media tools can contribute to both the enhancement and detriment of learners’ personalities. Adequate professional development of faculty members or teachers as authentic leaders can assist students in enhancing their learning experiences beyond the classroom walls using social media tools. Teachers, acting as authentic leaders, can also guide other school staff, such as counselors, school librarians, and coordinators, in addressing students’ privacy and intimacy concerns in physical and virtual spaces.

The evolution of teachers into authentic leaders through social media-based learning initiates a sequence of events. Initially, teachers incorporate social media-based learning into their practices. Subsequently, they lead with authenticity in the realm of social media. Ultimately, they nurture their students as authentic leaders through social media-based learning. Failing to provide guidance on appropriate sharing and responsible online behavior places students at significant risk. The convenience, flexibility, and widespread use of social media for content sharing can become problematic if left unchecked. Anything shared by students in the digital realm becomes part of the global network (Boyd and Ellison, 2007).

Authentic leaders can cultivate conducive social media environments for learning and fostering student personalities. Firstly, they must comprehend the gravity of students’ educational, psychological, and social challenges in both physical and cyber spaces. Secondly, school staff, including counselors, coordinators, librarians, and principals, can devise a social media policy to nurture a well-rounded, unbiased, and inclusive student personality. Thirdly, teachers as authentic leaders can address pertinent educational issues. For instance, bullying is a pervasive concern that traditionally occurred during school hours. However, with social media, students face the threat of bullying around the clock. Teachers, assuming the role of authentic leaders, can establish guidelines for educational matters, such as students’ academic freedom and privacy rights on social media platforms. These social media networks can be expanded to the district and national levels to connect educational stakeholders, such as policymakers, curriculum developers, and teacher education departments, for updates on fieldwork.

## 6. Limitations and future work

This study possesses certain limitations that we propose to address in future research endeavors. Firstly, our study employed a simplified version of the technology acceptance model (TAM) to amalgamate two expansive theories, technology acceptance and authentic leadership. Subsequent research could encompass additional exogenous components as suggested by previous studies such as theory of planned behavior (Ajzen, 2020) components which includes attitude, subjective norms, and external variables.

Secondly, our investigation focused on the second-order factor of authentic leadership. In forthcoming studies, it might be advantageous to deconstruct authentic leadership into its constituent elements and seamlessly incorporate them into the TAM framework. As this study marked the initial endeavor to fuse TAM with authentic leadership development, there exists potential to incorporate information science theories into other social learning frameworks, such as the community of inquiry and leadership development. This integration could yield insights beneficial for higher education institutions, curriculum developers, and educational policymakers in embracing social media-based learning to foster the professional growth of educators.

## Data availability statement

The original contributions presented in the study are included in the article/supplementary material, further inquiries can be directed to the corresponding authors.

## Ethics statement

The research design was approved by the ethics committee of the Open University of Catalonia, Barcelona University, and the University of Management and Technology, Lahore, Pakistan. During the two surveys, the process remained uniform, and reliable tools were used to conduct the research. The participation was voluntary, and it was kept confidential. Each participant was informed about the purpose of the study and assured of anonymity. The participants signed an informed consent letter before completing the questionnaire.

## Author contributions

MZA: conceptualization, data curation, formal analysis, methodology, and writing—original draft. SFR: conceptualization and validation. PS-H, SA, and SB: methodology and validation. PS-H: funding acquisition, conceptualization and supervision. All authors contributed to the article and approved the submitted version.

## References

[ref1] Acarli D. S. Sağlam Y. (2015). Investigation of pre-service teachers’ intentions to use of social Media in Teaching Activities within the framework of technology acceptance model. Procedia Soc. Behav. Sci. 176, 709–713. doi: 10.1016/j.sbspro.2015.01.530

[ref2] Ajzen I. (2020). The theory of planned behavior: frequently asked questions. Hum. Behav. Emerg. Technol. 2, 314–324. doi: 10.1002/hbe2.195

[ref3] Alaimo C. Kallinikos J. (2019). “Social media and the infrastructuring of sociality” in Research in the Sociology of Organizations. ed. Lawle E. J. (Bingley, UK: Emerald Group Publishing).

[ref4] Alsaleh D. A. Elliott M. T. Fu F. Q. Thakur R. (2019). Cross-cultural differences in the adoption of social media. J. Res. Interact. Mark. 13, 119–140. doi: 10.1108/JRIM-10-2017-0092

[ref5] Alshurideh M. Salloum S. A. Al Kurdi B. Monem A. A. Shaalan K. (2019). Understanding the quality determinants that influence the intention to use the mobile learning platforms: a practical study. Int. J. Interact. Mob. Technol. 13:10300. doi: 10.3991/ijim.v13i11.10300

[ref6] Asghar M. Z. Arif S. Barbera E. Seitamaa-Hakkarainen P. Kocayoruk E. (2021). Support through social media and online class participation to enhance psychological resilience. Int. J. Environ. Res. Public Health 18:11962. doi: 10.3390/ijerph182211962, PMID: 34831716 PMC8624183

[ref7] Asghar M. Z. Arif S. Iqbal J. Seitamaa-Hakkarainen P. (2022). Social media tools for the development of pre-service health sciences researchers during COVID-19 in Pakistan. Int. J. Environ. Res. Public Health 19:581. doi: 10.3390/ijerph19010581, PMID: 35010843 PMC8744709

[ref8] Asghar M. Z. Barbera E. Rasool S. F. Seitamaa-Hakkarainen P. Mohelská H. (2022). Adoption of social media-based knowledge-sharing behaviour and authentic leadership development: evidence from the educational sector of Pakistan during COVID-19. J. Knowl. Manag. 27, 59–83. doi: 10.1108/JKM-11-2021-0892

[ref9] Asghar M. Z. Barberà E. Younas I. (2021). Mobile learning technology readiness and acceptance among pre-service teachers in Pakistan during the COVID-19 pandemic. Knowl. Manage. E Learn. 13, 83–101. doi: 10.34105/j.kmel.2021.13.005

[ref10] Asghar M. Z. Erdoğmuş Y. K. Seitamaa-Hakkarainen P. (2021). Cultural levels and pre-service teachers’ behaviour towards the use of open educational resources. J. Interact. Media Educ. 2021, 1–21. doi: 10.5334/jime.674

[ref11] Asghar M. Z. Iqbal A. Seitamaa-Hakkarainen P. Barbera E. (2021). Breaching learners’ social distancing through social media during the covid-19 pandemic. Int. J. Environ. Res. Public Health 18:1012. doi: 10.3390/ijerph182111012, PMID: 34769534 PMC8583489

[ref12] Asghar M. Z. Iqbal J. Seitamaa-Hakkarainen P. Barbera E. Ozbilen F. M. Waqar Y. (2023). Symmetrical and asymmetrical modeling: applying vitae researchers’ development framework through the Lens of web 2.0 Technologies for Vocational-Health Education Researchers. Sustainability 15:7514. doi: 10.3390/su15097514

[ref13] Assiri M. (2018). School leaders’ practice of the ethics of educational leadership to make decisions. Journal of education in Black Sea. Region 4, 10–34. doi: 10.31578/jebs.v4i1.151

[ref14] Avolio B. J. Gardner W. L. (2005). Authentic leadership development: getting to the root of positive forms of leadership. Leadersh. Q. 16, 315–338. doi: 10.1016/j.leaqua.2005.03.001

[ref15] Balogun T. Mahembe B. Allen-Ile C. (2020). A confirmatory factor analytic study of an authentic leadership measure in Nigeria. SA journal of. Hum. Resour. Manag. 23:18. doi: 10.4102/sajhrm.v18i0.1235

[ref16] Beddoes-Jones F. Swailes S. (2015). Authentic leadership: development of a new three pillar model. Strateg. HR Rev. 14, 94–99. doi: 10.1108/SHR-04-2015-0032

[ref17] Behringer N. Sassenberg K. (2015). Introducing social media for knowledge management: determinants of employees’ intentions to adopt new tools. Comput. Hum. Behav. 48, 290–296. doi: 10.1016/j.chb.2015.01.069

[ref18] Bertoncini G. T. Schmalz M. T. (2013). What’s on your mind? Understanding the influence of social media on authentic leadership dimensions and education from the millennials’ perspective. Kalmar, Sweeden: Linnaeus University.

[ref19] Boyd D. M. Ellison N. B. (2007). Social network sites: definition, history, and scholarship. Journal of computer-mediated. Communication 13, 210–230. doi: 10.1111/j.1083-6101.2007.00393.x

[ref20] Bryan V. Vitello-Cicciu J. (2022). Perceptions of preceptors’ authentic leadership and final year nursing students’ self-efficacy, job satisfaction, and job performance. J. Prof. Nurs. 41, 81–87. doi: 10.1016/j.profnurs.2022.04.003 35803664

[ref21] Chen F. F. (2007). Sensitivity of goodness of fit indexes to lack of measurement invariance. Struct. Equ. Modeling 14, 464–504. doi: 10.1080/10705510701301834

[ref22] Cohen J. (1988). Statistical power analysis Jbr the behavioral. Sciences Hillsdale, NJ: Lawrence Erlbaum Associates, pp. 18–74.

[ref23] Corriveau A. M. (2020). Developing authentic leadership as a starting point to responsible management: a Canadian university case study. International. J. Manag. Educ. 18:100364. doi: 10.1016/j.ijme.2020.100364

[ref24] Daniëls E. Hondeghem A. Dochy F. (2019). A review on leadership and leadership development in educational settings. Educ. Res. Rev. 27, 110–125. doi: 10.1016/j.edurev.2019.02.003

[ref25] Davis F. D. (1989). Perceived usefulness, perceived ease of use, and user acceptance of information technology. MIS Q. 13:319. doi: 10.2307/249008

[ref26] Deffenbaugh D. G. (2010). Born digital: understanding the first generation of digital natives - by John palfrey and Urs gasser. Teach. Theol. Relig. 13, 381–384. doi: 10.1111/j.1467-9647.2010.00655.x

[ref27] Demers J. A. Sullivan A. L. (2016). Confronting the ubiquity of electronic communication and social media: ethical and legal considerations for psychoeducational practice. Psychol. Sch. 53, 517–532. doi: 10.1002/pits.21920

[ref28] Doleck T. Bazelais P. Lemay D. J. (2017). Examining the antecedents of social networking sites use among CEGEP students. Educ. Inf. Technol. 22, 2103–2123. doi: 10.1007/s10639-016-9535-4

[ref29] Duh H. I. Dabula N. (2021). Millennials’ socio-psychology and blood donation intention developed from social media communications: a survey of university students. Telematics Inform. 58:101534. doi: 10.1016/j.tele.2020.101534

[ref30] Dumpit D. Z. Fernandez C. J. (2017). Analysis of the use of social media in higher education institutions (HEIs) using the technology acceptance model. Int. J. Educ. Technol. High. Educ. 14:45. doi: 10.1186/s41239-017-0045-2

[ref31] El-Gayar O. F. Moran M. (2006). *College students’ acceptance of tablet PCs: an application of the UTAUT model*. In: 36th annual meeting of the decision sciences institute (DSI).

[ref32] Fiss P. C. (2011). Building better causal theories: a fuzzy set approach to typologies in organization research. Acad. Manag. J. 54, 393–420. doi: 10.5465/amj.2011.60263120

[ref33] Gardner H. (2011). *Multiple intelligences: reflections after thirty years*. Washington, DC: National Association of Gifted Children Parent and Community Network Newsletter.

[ref34] Gardner W. L. Avolio B. J. Luthans F. May D. R. Walumbwa F. (2005). “Can you see the real me?” a self-based model of authentic leader and follower development. Leadersh. Q. 16, 343–372. doi: 10.1016/j.leaqua.2005.03.003

[ref35] George B. (2003). Authentic leadership: rediscovering the secrets to creating lasting value. 250. New York: The Publishers Weekly.

[ref36] Grimsey D. Lewis M. K. (2002). Evaluating the risks of public private partnerships for infrastructure projects. Int. J. Proj. Manag. 20, 107–118. doi: 10.1016/S0263-7863(00)00040-5

[ref37] Hair J. F. Sarstedt M. Matthews L. M. Ringle C. M. (2016). Identifying and treating unobserved heterogeneity with FIMIX-PLS: part I–method. Eur. Bus. Rev. 28, 63–76. doi: 10.1108/EBR-09-2015-0094

[ref38] Hair J. F. Ringle C. M. Sarstedt M. (2011). PLS-SEM: indeed a silver bullet. J. Mark. Theory Pract. 19, 139–152. doi: 10.2753/MTP1069-6679190202

[ref39] Hair J. F. Ringle C. M. Sarstedt M. (2013). Partial least squares structural equation modeling: rigorous applications, better results and higher acceptance. Long Range Plan. 46, 1–12. doi: 10.1016/j.lrp.2013.01.001

[ref40] Hall P. Jing B. (1996). On sample reuse methods for dependent data. J. R. Stat. Soc. B Methodol. 58, 727–737. doi: 10.1111/j.2517-6161.1996.tb02111.x

[ref41] Hendriks M. Martijn B. Antoinette R. Emma P. Harry C. (2020). Virtuous leadership and employee flourishing: the mediating role of work engagement. Manus. Publ. 2, 1–39.

[ref42] Henseler J. (2012). “PLS-MGA: a non-parametric approach to partial least squares-based multi-group analysis” in Studies in classification, data analysis, and knowledge organization. eds. Gaul W. A. Geyer-Schulz A. Schmidt-Thieme L. (Berlin: Springer)

[ref43] Hu L. Bentler P. M. (1998). Fit indices in covariance structure modeling: sensitivity to underparameterized model misspecification. Psychol. Methods 3, 424–453. doi: 10.1037/1082-989X.3.4.424

[ref44] Hussain M. Rasool S. F. Xuetong W. Asghar M. Z. Alalshiekh A. S. A. (2023). Investigating the nexus between critical success factors, supportive leadership, and entrepreneurial success: evidence from the renewable energy projects. Environ. Sci. Pollut. Res. 30, 49255–49269. doi: 10.1007/s11356-023-25743-w, PMID: 36764994

[ref45] Ifinedo P. (2018). Determinants of students’ continuance intention to use blogs to learn: an empirical investigation. Behav. Inf. Technol. 37, 381–392. doi: 10.1080/0144929X.2018.1436594

[ref46] Iszatt-White M. Carroll B. Gardiner R. Kempster S. (2019). Leadership special Issue: do we need authentic leadership? Interrogating authenticity in a new world order. Leadership 15:269. doi: 10.1177/1742715019855269

[ref47] Jacob J. Pattusamy M. (2020). Examining the inter-relationships of UTAUT constructs in mobile internet use in India and Germany. J. Electron. Commer. Organ. 18, 36–48. doi: 10.4018/JECO.2020040103

[ref48] Johnson D. G. (2015). Technology with no human responsibility? J. Bus. Ethics 127, 707–715. doi: 10.1007/s10551-014-2180-1

[ref49] Kemp S. (2021a). *Digital 2021: global overview report–Data Reportal–global digital insights*. Kepios Pte. Ltd., We Are Social Ltd., Hootsuite Inc.

[ref50] Kemp S. (2021b). *Digital 2021: Pakistan*. Available at: https://datareportal.com/reports/digital-2021-pakistan.

[ref51] Khan M. M. S. Ghayas M. M. (2022). Impact of authentic leadership on employee engagement in the banking sector of Karachi. Int. J. Bus. Perform. Manag. 23:90. doi: 10.1504/IJBPM.2022.10042798

[ref52] Kietzmann J. H. Silvestre B. S. Mccarthy I. P. Pitt L. F. (2012). Unpacking the social media phenomenon: towards a research agenda. J. Public Aff. 12, 109–119. doi: 10.1002/pa.1412

[ref53] Kundu A. Bej T. (2021). We have efficacy but lack infrastructure: teachers’ views on online teaching learning during COVID-19. Qual. Assur. Educ. 29, 344–372. doi: 10.1108/QAE-05-2020-0058

[ref54] Liu K. Tao D. (2022). The roles of trust, personalization, loss of privacy, and anthropomorphism in public acceptance of smart healthcare services. Comput. Hum. Behav. 127:107026. doi: 10.1016/j.chb.2021.107026

[ref55] Liu S. Zaigham G. H. K. Rashid R. M. Bilal A. (2022). Social media-based collaborative learning effects on student performance/learner performance with moderating role of academic self-efficacy. Front. Psychol. 13:13. doi: 10.3389/fpsyg.2022.903919 PMC9309218 35899006

[ref56] Loon M. Evans J. Kerridge C. (2015). Learning with a strategic management simulation game: a case study. International. J. Manag. Educ. 13, 227–236. doi: 10.1016/j.ijme.2015.06.002

[ref57] Magsamen-Conrad K. Wang F. Tetteh D. Lee Y. I. (2020). Using technology adoption theory and a lifespan approach to develop a theoretical framework for eHealth literacy: extending UTAUT. Health Commun. 35, 1435–1446. doi: 10.1080/10410236.2019.1641395, PMID: 31328567

[ref58] McLoughlin C. Lee M. J. W. (2007). *Social software and participatory learning: pedagogical choices with technology affordances in the web 2.0 era*. In: ASCILITE 2007 - the Australasian Society for Computers in learning in tertiary education.

[ref59] McLoughlin C. Lee M. J. W. (2010). Personalised and self regulated learning in the web 2.0 era: international exemplars of innovative pedagogy using social software. Australas. J. Educ. Technol. 26:1100. doi: 10.14742/ajet.1100

[ref60] Mohammadi H. (2015). Investigating users’ perspectives on e-learning: an integration of TAM and IS success model. Comput. Hum. Behav. 45, 359–374. doi: 10.1016/j.chb.2014.07.044

[ref61] Na S. Heo S. Han S. Shin Y. Roh Y. (2022). Acceptance model of artificial intelligence (AI)-based Technologies in Construction Firms: applying the technology acceptance model (TAM) in combination with the technology–organisation–environment (TOE) framework. Buildings 12:90. doi: 10.3390/buildings12020090

[ref62] Nair B. P. Prasad T. Nair S. K. (2021). Self-awareness trigger leading to authentic leadership: conceptualization and development of reliable and valid self-awareness trigger scale. Dyn. Relat. Manage. J. 10:2. doi: 10.17708/DRMJ.2021.v10n01a02

[ref63] Olya H. Jung T. H. Tom Dieck M. C. Ryu K. (2020). Engaging visitors of science festivals using augmented reality: asymmetrical modelling. Int. J. Contemp. Hosp. Manag. 32, 769–796. doi: 10.1108/IJCHM-10-2018-0820

[ref64] Pappas N. Papatheodorou A. (2017). Tourism and the refugee crisis in Greece: perceptions and decision-making of accommodation providers. Tour. Manag. 63, 31–41. doi: 10.1016/j.tourman.2017.06.005

[ref65] Pappas I. O. Woodside A. G. (2021). Fuzzy-set qualitative comparative analysis (fsQCA): guidelines for research practice in information systems and marketing. Int. J. Inf. Manage. 58:102310. doi: 10.1016/j.ijinfomgt.2021.102310

[ref66] Popescu E. Badea G. (2020). Exploring a community of inquiry supported by a social media-based learning environment. Educ. Technol. Soc. 23, 61–76.

[ref67] Proserpio L. Gioia D. A. (2007). Teaching the virtual generation. Acad. Manage. Learn. Educ. 6, 69–80. doi: 10.5465/amle.2007.24401703

[ref68] Ragin C. C. (2008). “Measurement versus calibration: A set-theoretic approach” in The Oxford Handbook of Political Methodology. eds. Box-Steffensmeier J. M. Brady H. E. Collier D. (Oxford, UK: Oxford University Press).

[ref69] Rasool S. F. Wang M. Tang M. Saeed A. Iqbal J. (2021). How toxic workplace environment effects the employee engagement: the mediating role of organizational support and employee wellbeing. Int. J. Environ. Res. Public Health 18:2294. doi: 10.3390/ijerph18052294, PMID: 33652564 PMC7956351

[ref70] Rasool S. F. Wang M. Zhang Y. Samma M. (2020). Sustainable work performance: the roles of workplace violence and occupational stress. Int. J. Environ. Res. Public Health 17:912. doi: 10.3390/ijerph17030912, PMID: 32024195 PMC7037902

[ref71] Rasoolimanesh S. M. Ringle C. M. Sarstedt M. Olya H. (2021). The combined use of symmetric and asymmetric approaches: partial least squares-structural equation modeling and fuzzy-set qualitative comparative analysis. Int. J. Contemp. Hosp. Manag. 33, 1571–1592. doi: 10.1108/IJCHM-10-2020-1164

[ref72] Rubinson C. (2019). Presenting qualitative comparative analysis: notation, tabular layout, and visualization. Method Innov. 12:6211. doi: 10.1177/2059799119862110

[ref73] Salas E. Wildman J. Piccolo R. (2009). Using simulation-based training to enhance management education. Acad. Manage. Learn. Educ. 8, 559–573. doi: 10.5465/AMLE.2009.47785474

[ref74] Samma M. Zhao Y. Rasool S. F. Han X. Ali S. (2020). Exploring the relationship between innovative work behavior, job anxiety, workplace ostracism, and workplace incivility: empirical evidence from small and medium sized enterprises (SMEs). Healthcare. 8:508. doi: 10.3390/healthcare8040508, PMID: 33238510 PMC7711530

[ref75] Sarstedt M. Ringle C. M. Smith D. Reams R. Hair J. F. (2014). Partial least squares structural equation modeling (PLS-SEM): a useful tool for family business researchers. J. Fam. Bus. Strat. 5, 105–115. doi: 10.1016/j.jfbs.2014.01.002

[ref76] Sholihin M. Sari R. C. Yuniarti N. Ilyana S. (2020). A new way of teaching business ethics: the evaluation of virtual reality-based learning media. International. J. Manag. Educ. 18:100428. doi: 10.1016/j.ijme.2020.100428

[ref77] Sophea D. Sungsuwan T. Viriyasuebphong P. (2022). Factors influencing students’ behavioral intention on using mobile learning (M-learning) in tourism and hospitality major in Phnom Penh, Cambodia. Curr Appl. Sci. Technol. 22:10. doi: 10.55003/cast.2022.02.22.010

[ref78] Steffens N. K. Wolyniec N. Okimoto T. G. Mols F. Haslam S. A. Kay A. A. (2021). Knowing me, knowing us: personal and collective self-awareness enhances authentic leadership and leader endorsement. Leadersh. Q. 32:101498. doi: 10.1016/j.leaqua.2021.101498

[ref79] Stoller E. (2013). Our shared future: social media, leadership, vulnerability, and digital identity. J. Coll. Character 14, 5–10. doi: 10.1515/jcc-2013-0002

[ref80] Surendran P. (2012). Technology acceptance model: a survey of literature. Int. J. Bus. Soc. Res. 2:161. doi: 10.18533/ijbsr.v2i4.161

[ref81] Tuten T. Mintu-Wimsatt A. (2018). Advancing our understanding of the theory and practice of social media marketing: introduction to the special ISSUE. J. Mark. Theory Pract. 26, 1–3. doi: 10.1080/10696679.2018.1393277

[ref82] Tuten T. Solomon M. Ladik D. (2015). *The teaching of social media marketing*. In: Developments in Marketing Science: Proceedings of the Academy of Marketing Science.

[ref83] Venkat Raman G. Garg S. Thapliyal S. (2019). Integrative live case: a contemporary business ethics pedagogy. J. Bus. Ethics 155, 1009–1032. doi: 10.1007/s10551-017-3514-6

[ref84] Venkatesh V. Morris M. G. Davis G. B. Davis F. D. (2003). User acceptance of information technology: toward a unified view. MIS Q. 27, 425–478. doi: 10.2307/30036540

[ref85] Voshel E. H. Wesala A. (2015). Social media & social work ethics: determining best practices in an ambiguous reality. J. Soc. Work Values Ethics 12, 67–76.

[ref86] Vuković M. Pivac S. Kundid D. (2019). Technology acceptance model for the internet banking acceptance in Split. Business. Syst. Res. 10, 124–140. doi: 10.2478/bsrj-2019-022

[ref87] Wang L. C. Calvano L. (2015). Is business ethics education effective? An analysis of gender, personal ethical perspectives, and moral judgment. J. Bus. Ethics 126, 591–602. doi: 10.1007/s10551-013-1973-y

[ref88] Wankel C. (2009). Management education using social media. Organ. Manage. J. 6, 251–262. doi: 10.1057/omj.2009.34

[ref89] Warnick B. R. Bitters T. A. Falk T. M. Kim S. H. (2016). Social media use and teacher ethics. Educ. Policy 30, 771–795. doi: 10.1177/0895904814552895

[ref90] White C. L. Boatwright B. (2020). Social media ethics in the data economy: issues of social responsibility for using Facebook for public relations. Public Relat. Rev. 46:101980. doi: 10.1016/j.pubrev.2020.101980

[ref91] Wijesundara T. R. Xixiang S. (2018). Social networking sites acceptance: the role of personal innovativeness in information technology. International. J. Bus. Manag. 13:75. doi: 10.5539/ijbm.v13n8p75

[ref92] Wolf M. Sims J. Yang H. (2017). *Social media? What social media? UK academy for information systems conference proceedings 2018*.

[ref93] Yurdakul I. K. Ursavaş Ö. F. Işçitürk G. B. (2014). An integrated approach for preservice teachers’ acceptance and use of technology: UTAUT-PST scale. Eurasian. J. Educ. Res. 55, 21–36. doi: 10.14689/ejer.2014.55.2

[ref94] Zaineldeen S. Hongbo L. Koffi A. L. Hassan B. M. A. (2020). Technology acceptance model’ concepts, contribution, limitation, and adoption in education. Universal. J. Educ. Res. 8, 5061–5071. doi: 10.13189/ujer.2020.081106

[ref95] Zhou X. Rasool S. F. Yang J. Asghar M. Z. (2021). Exploring the relationship between despotic leadership and job satisfaction: the role of self efficacy and leader–member exchange. Int. J. Environ. Res. Public Health 18:5307. doi: 10.3390/ijerph18105307, PMID: 34067634 PMC8155868

[ref96] Zimmerman B. J. (1990). Self-regulated learning and academic achievement: an overview. Educ. Psychol. 25, 3–17. doi: 10.1207/s15326985ep2501_2

